# The impact of digital transformation on faculty performance in higher education: the mediating role of digital self-efficacy and the moderating role of task-technology fit

**DOI:** 10.3389/fpsyg.2025.1693375

**Published:** 2025-10-20

**Authors:** TingYu Sun, ManKeun Yoon

**Affiliations:** ^1^Department of Labor Relations, Shandong Management University, Jinan, China; ^2^Department of Education, The Catholic University of Korea, Bucheon, Republic of Korea

**Keywords:** performance, digital transformation, digital self-efficacy, task-technology fit, higher education

## Abstract

Faculty performance is a fundamental driver of sustainable development in higher education institutions. In the era of Education 4.0, the digitalization of education has had a profound impact on the faculty work content and methods. To investigate how digital transformation impacts faculty performance in higher education, this study surveyed 482 university faculty members. The results of the study showed that digital transformation is a significant positive predictor of faculty performance. Digital self-efficacy partially mediates the relationship between digital transformation and faculty performance. Task-technology fit positively enhances the impact of digital transformation on faculty performance. When the level of task-technology fit is high, the indirect effect of digital transformation on faculty performance through digital self-efficacy is stronger.

## 1 Introduction

Teachers are vital resources in the education system, serving as primary implementers and practitioners of institutional missions. Their performance not only reflects the overall quality of higher education institutions but also serves as a prerequisite for high-quality educational development (Li et al., 2025). Teacher performance refers to all educational actions and activities taken by teachers to achieve school goals (Hwang et al., 2017), and is closely related to their outcomes (Chen et al., 2021; Fan, 2022; Unruh, 2024). Enhancing teacher performance not only improves student academic achievements but also stimulates educators’ professional enthusiasm and creativity, serving as an enduring driving force for institutional sustainability.

In recent years, academic research has actively explored factors influencing teacher performance. These include organizational elements such as principals’ leadership styles (Berhanu, 2025), school climate (Dutta and Sahney, 2021), motivational approaches (Durksen et al., 2017), and organizational culture (Durksen et al., 2017). At the individual level, key factors encompass teachers’ job satisfaction (Li et al., 2025), emotional intelligence (Lu and Chen, 2024), organizational commitment (Wayoi et al., 2021), and well-being (Amirian et al., 2023). Beyond traditional influencing factors, the rapid development of digital technologies in modern society has made digitization a crucial factor affecting teacher performance. Entering the era of Education 4.0 (Mukul and Büyüközkan, 2023; de Oliveira and de Souza, 2022), the digital transformation of education is unfolding globally (Mohamed Hashim et al., 2022).

Digital transformation, as an organizational-level intervention, primarily impacts core actors within educational institutions—teachers. By altering teachers’ tools, processes, and environments, it directly influences their teaching effectiveness, research efficiency, and professional practice. Consequently, this has prompted a series of studies examining the effects of educational digitization on teacher performance. Numerous existing studies have examined teachers’ technology adoption and acceptance (Granić, 2022; Lai et al., 2022). However, technology usage does not equate to successful digital transformation. The ultimate goal of transformation lies in enhancing the effectiveness and quality of teachers’ work. Although digital transformation may also impact student learning performance or organizational performance, these outcomes are predicated upon faculty performance, which serves as the cornerstone for other performance metrics within higher education institutions. Therefore, examining the effects of digital transformation on faculty performance represents the shortest and most direct pathway. Understanding this influence pathway is a logical prerequisite for comprehending its ultimate impact on students and organizations. Our research focuses on this phenomenon by investigating how a major organizational transformation (digital transformation) is internalized and transformed by faculty members, ultimately manifesting as improvements in their daily work performance.

Despite the prevailing trend of digital transformation, the actual integration of digital technologies in educational practice remains limited. A survey shows that only 41% of teachers use digital technology in their teaching (Drossel et al., 2019), and factors such as insufficient training (ElSayary, 2023), lack of digital infrastructure (Blaskó et al., 2022), and low willingness to adopt digital technologies (Spiteri and Chang Rundgren, 2020) are the main reasons for its ineffective integration. All these phenomena are the result of an immature stage of digital transformation. Unlike in primary and secondary schools, faculty work in higher education encompasses teaching, research, administration, and social services (Lashuel, 2020), making the process of digital transformation more complex. Moreover, as a bridge between schools and society, universities produce a wide range of professionals for society, making it especially critical to translate digital technologies into faculty performance and to cultivate talent capable of adapting to the needs of the current digital industry (Akour and Alenezi, 2022).

According to the available research, most existing studies focus on the impact of a particular digital technology on faculty work. While valuable, this approach fails to capture the full landscape of educational digital transformation—an integrated ecological shift driven by the convergence of multiple technologies that systematically reshapes educational philosophies, models, and processes (Mercader and Gairín, 2020). Therefore, we conceptualize digital transformation as a higher-order, multidimensional construct to examine its overall impact on faculty performance. This approach helps shift academic discourse from debating “whether a specific technology is effective” to exploring “how a digital ecosystem influences education,” thereby complementing existing research perspectives. Furthermore, existing research predominantly examines the superficial relationship between technological factors and performance, and lacks an in-depth analysis of the mediating and moderating mechanisms underlying this relationship. Our study not only focuses on direct effects but also seeks to uncover the underlying processes and relevant boundary conditions of this influence, thereby enhancing the explanatory power and contextualization of the impact pathways.

In order to study the impact of digital transformation on faculty performance in higher education and its intrinsic mechanism, self-determination theory is introduced in this study. According to self-determination theory, human beings are born with the basic psychological needs of competence, relatedness and autonomy, and the realization of these needs is affected by the external environment. When the environment is able to satisfy the basic psychological needs, it increases the individual’s behavioral motivation and the quality of engagement, such as performance and creativity (Deci and Ryan, 2013). Therefore, the external environment of digital transformation may affect faculty performance by influencing their psychological needs. Self-efficacy is the core element of the psychological need for “competence,” which refers to an individual’s subjective assessment, judgment, and prediction of whether or not they can successfully perform a task or achievement (Bandura, 1977). Faculty digital self-efficacy (DSE), by extension, refers to their confidence in successfully utilizing digital technology to perform tasks in digital environments, which largely determines whether faculty use digital technology and how they use it, and contributes to their digital competence (Ulfert-Blank and Schmidt, 2022). Research has found that mature digital transformation initiatives (e.g., digital facilities, digital climate, leadership support) help teachers gain successful experiences in technology adoption, thereby enhancing their digital self-efficacy (Omar and Ismail, 2021). Moreover, digital self-efficacy has been shown to help users utilize technology more effectively (Paredes-Aguirre et al., 2024), thereby more effectively leveraging the role of digital technology in promoting teacher performance. Therefore, this study aims to explore whether digital self-efficacy plays a mediating role between digital transformation and faculty performance.

Furthermore, to better leverage digital technology in higher education settings to enhance faculty performance, this study attempts to identify the boundary conditions under which digital transformation affects faculty performance. The Task-Technology Fit (TTF) theory integrates technological efficiency with task requirements, personnel attributes, and organizational contexts, proposing that technology effectiveness largely depends on its alignment with specific tasks. A higher degree of fit between technology and tasks correlates with improved efficiency and outcomes, which positively affects individual performance (Goodhue and Thompson, 1995). In higher education institutions, faculty from different disciplines have different tasks, and the digital environments they work in and the digital technologies they use also vary (Nkomo et al., 2021), resulting in varying degrees of task-technology fit. Research reveals that arts and humanities faculty face the greatest barriers in integrating digital technologies (Mercader and Gairín, 2020). Therefore, there may also be differences in the impact of digital transformation on faculty performance in the context of disciplinary differentiation within higher education institutions. This study aims to examine the conditions that should be in place for task technology fit in the impact of digital transformation on teacher performance in higher education.

This study not only reveals the underlying mechanisms linking digital transformation to faculty performance, aiding institutional administrators and faculty in translating digital technologies into improved performance more efficiently, but also identifies boundary conditions providing higher education with more specific and targeted guidance for implementing digital transformation across diverse contexts. This research offers robust support for universities seeking to optimize digital talent development. Building on this, the purpose of this study is to investigate the impact of digital transformation in higher education on faculty performance, validate the mediating role of digital self-efficacy, and identify the moderating role of task-technology fit, thereby facilitating the efficient translation of digital technology into faculty performance. The main questions addressed in this study are:

1. Does digital transformation in higher education institutions affect faculty performance?
2. Can digital transformation in higher education affect faculty performance through digital self-efficacy?
3. Does task-technology fit serve as a boundary condition for the impact of digital transformation on faculty performance in higher education institutions?

## 2 Theoretical background and research hypotheses

### 2.1 Digital transformation and faculty performance

Digitalization refers to the use of digital technology to improve processes and convert data into information (Marks and AL-Ali, 2022). Digital transformation, also referred to as digital maturity, denotes an organization’s achieved level and sophistication in digitalization (Begicevic Redjep et al., 2021). It is closely related to the development of information and communication technology (Vyas-Doorgapersad, 2022). Digital transformation is a key component of the future sustainable strategy for the global higher education sector, and universities must leverage it as a driving force to build competitive advantages for themselves (Mohamed Hashim et al., 2022). Educational digital transformation requires educational organizations to review their strategies to integrate digital technologies into their teaching, learning, and management practices and become digitally mature institutions. Its main components include planning, management, and leadership; ICT in teaching and learning; development of digital competences; ICT culture; and ICT infrastructure (Begicevic Redjep et al., 2021).

Teacher performance is a key indicator for measuring educational quality and organizational effectiveness. In existing research, its definition can generally be categorized into two perspectives: behavioral (Ali and Haider, 2017; Hwang et al., 2017) and outcome (Jamal, 2007; Motowidlo, 2003). In this study, faculty performance (FP) is explored from a behavioral perspective and refers to all educational actions and activities undertaken by faculty to achieve the goals of higher education institutions (Hwang et al., 2017). Due to the complexity of the work of faculty, the concept of faculty performance is multidimensional, encompassing teaching, research, social services (Adeyemi, 2008), administrative management, talent cultivation, learning and growth, and professional ethics (Ali and Haider, 2017). In terms of influencing factors, faculty performance is believed to be influenced by the combined effects of individual characteristics (such as skills and motivation) and organizational environment (such as leadership and incentive mechanisms) (Kanya et al., 2021).

Digital transformation can influence faculty performance as an external environmental factor. In the corporate sector, numerous studies have confirmed the positive impact of digital transformation on performance (Peng and Tao, 2022; Teng et al., 2022; Zhai et al., 2022). Research also indicates that digitalization fosters greater employee accountability by enhancing job autonomy, self-efficacy, and peer closeness (Liu and Cheng, 2025). Within the education sector, digital transformation affects various dimensions of teacher performance. In terms of teaching, the diversification and personalization of digital resources can help students progress faster and more efficiently (Dhawan and Batra, 2021). Regarding research, technology has brought about efficient data collection and processing, smooth communication among members, and easier interdisciplinary research (Pisica et al., 2023). At the administrative level, it reduces repetitive tasks while improving the efficiency and security of administrative work (Pisica et al., 2023). Empirical studies have confirmed the positive impact of digital transformation on work performance and teaching outcomes. However, other studies indicate that the use of educational technology can induce anxious and stress among teachers, thereby impairing their mental health and work quality (Fernández-Batanero et al., 2021). Nevertheless, the negative impact is smaller for teachers who are already familiar with new technologies and those whose institutions are equipped with comprehensive technological tools and facilities (Fütterer et al., 2023; Wohlfart et al., 2021). It can therefore be concluded that educational organizations with a high level of digital transformation maturity are better equipped to mitigate the negative ways in which digital technologies affect faculty. Therefore, this study aims to examine how the digital transformation environment affects faculty performance within the context of higher education reform. Based on this, the following hypothesis is proposed:

*H1*: Digital transformation in higher education institutions can positively promote faculty performance.

### 2.2 Self-determination theory and the mediating role of digital self-efficacy

Self-determination theory, proposed by Deci and Ryan (2013), posits that individual behavior is driven by the fulfillment of three basic psychological needs: autonomy, competence, and relatedness. ‘Autonomy’ refers to the perception that behavior is self-initiated and consistent with one’s values; ‘Competence’ refers to confidence in effectively coping with environmental challenges; and ‘Relatedness’ refers to the need to connect with others. Self-determination refers to the free choice of actions made by individuals based on a thorough understanding of their own needs and the social environment (Leroy et al., 2015). According to self-determination theory, the degree to which individuals exercise self-determination over their own behavior is influenced by the support provided by the external environment. In other words, when the external environment supports the fulfillment of an individual’s basic psychological needs, the individual internalizes external rules and values, forming autonomous internal motivation. At this point, the individual’s behavior also exhibits a higher degree of self-determination (Ryan and Deci, 2000).

Digital self-efficacy refers to self-efficacy related to digital technology. Self-efficacy is an individual’s belief in successfully performing specific tasks (Bandura, 1977) and is considered closely related to personal performance (Cherian and Jacob, 2013). From the perspective of self-determination theory, self-efficacy is a specific manifestation of the basic psychological need for “competence” and a core element of intrinsic motivation. High self-efficacy directly satisfies the need for competence, thereby promoting intrinsic motivation and psychological well-being. In a digital context, digital self-efficacy refers to an individual’s confidence in successfully using digital technologies. It is a core factor in predicting digital competence and technology acceptance, and it determines an individual’s use and adoption of digital technologies (Ulfert-Blank and Schmidt, 2022).

As a form of external environmental support, digital transformation can foster the development of digital self-efficacy. Research indicates that digital transformation has introduced mature digital devices and software, lowering the barrier to entry for teachers using digital technologies (Yu et al., 2017). Secondly, digital transformation integrates training on digital technology usage and digital competency into the teacher training system. This enhances teachers’ knowledge and technical proficiency, particularly in terms of technology integration and the use of digital resources, thereby reducing the technical stress new technologies impose on teachers (Instefjord and Munthe, 2017; Reisoğlu, 2022). At the same time, the digital transformation requires organizations to foster a digital culture that creates a positive, high-tolerance environment for digital use (Velyako and Musa, 2024). It can be seen that digital transformation affects faculty members’ self-determination by establishing a supportive digital environment that meets their fundamental psychological needs. This supportive environment encourages faculty to actively engage with technology and helps them acquire successful experiences, while enactive mastery experiences and vicarious experiences serve as powerful sources of self-efficacy (Bandura, 1977). Observing others’ successes as well as their own can enhance confidence in using digital technologies (Achterkamp et al., 2015). Therefore, we believe that digital transformation contributes to the development of faculty’s digital self-efficacy.

Digital self-efficacy positively influences faculty performance. Self-efficacy is closely related to individual performance (Saleh, 2008) and serves as a significant predictor of motivation and outcomes (Chang et al., 2014; Kapucu and Bahçivan, 2015). Existing research confirms that teachers’ self-efficacy not only enhances students’ academic achievement (Klassen and Tze, 2014) but also promotes their own teaching quality and instructional performance (Holzberger et al., 2013; Klassen and Tze, 2014). In the digital age, digital self-efficacy has a powerful impact on teachers’ behavior and achievement. It determines whether teachers will use technology, how they will use it, the extent to which they will use it, and the degree of success they will achieve in using it (Josip et al., 2022). Teachers’ digital self-efficacy and positive attitudes serve as reliable indicators for their integration of technology into the classroom (Cosby et al., 2023), with higher self-efficacy leading to more effective integration (Gomez et al., 2022). Therefore, we believe that digital self-efficacy can enhance faculty’s confidence in using digital technology, facilitate better integration of digital technology, and effectively translate digital technology into improved faculty performance.

Overall, digital transformation can promote the development of faculty members’ digital self-efficacy by helping them gain successful digital technology experiences. Furthermore, digital self-efficacy facilitates effective technology integration and successful outcomes, thereby enhancing faculty performance. Based on this, the following hypothesis is proposed:

*H2*: Digital self-efficacy plays a mediating role in the impact of digital transformation in higher education institutions on faculty performance.

### 2.3 Task-technology fit theory and the moderating role of task-technology fit

The Task-Technology Fit (TTF) theory was first proposed by Goodhue and Thompson (1995) to explain how information technology can enhance individual or organizational performance by aligning with task requirements. The theory posits that technology use is a necessary but insufficient condition for performance improvement. Adopting technology does not necessarily lead to improved performance. Only when the features of technology align with task requirements does technology use contribute to enhanced performance. The higher the degree of alignment between technology features and task requirements, the more beneficial technology use is for performance improvement (Goodhue and Thompson, 1995). The core components of the TTF theory encompass task characteristics, technology characteristics, task-technology fit, technology use, and performance impact (Goodhue and Thompson, 1995). Task-Technology Fit refers to the degree of alignment between technology and task requirements when individuals use technology to complete tasks. In the field of education, TTF theory emphasizes that technology should be matched with the specific needs and learning activities of teachers (students) (Zhang et al., 2025).

Task-technology fit affects the willingness and outcome of technology use (Dahri et al., 2024). Research has found that when task-technology fit is high, a mature digital transformation can enhance users’ perceived ease of use, perceived usefulness, and satisfaction with usage. These positive perceptions, in turn, enhances user experience and productivity, thereby promoting performance improvement (Yuce et al., 2019). This not only promotes performance improvement, but successful technological experiences also reinforce digital self-efficacy. Conversely, if technology is poorly suited to the task, forcing its application can increase cognitive load, leading to reduced efficiency or effectiveness (Buchner et al., 2022). Implementing digital transformation under such circumstances risks being perceived by teachers as an additional burden or administrative mandate, leading to a situation where the more advanced the technology becomes, the greater the anxiety among educators (Henderson and Corry, 2021). This not only undermines faculty performance but also fails to foster digital self-efficacy. Moreover, the frustration and apprehension stemming from technological pressures can further erode teachers’ digital self-efficacy (Li and Xie, 2025). Therefore, we believe that task-technology fit determines to some extent whether digital transformation is an enabling factor or a burden for faculty. Task-technology fit influences the effects of digital transformation on both faculty performance and digital self-efficacy. Based on this, the following hypotheses are proposed:

*H3a*: Task-technology fit moderates the impact of digital transformation in higher education on faculty performance.

*H3b*: Task-technology fit moderates the impact of digital transformation in higher education on digital self-efficacy.

**Figure 1:**
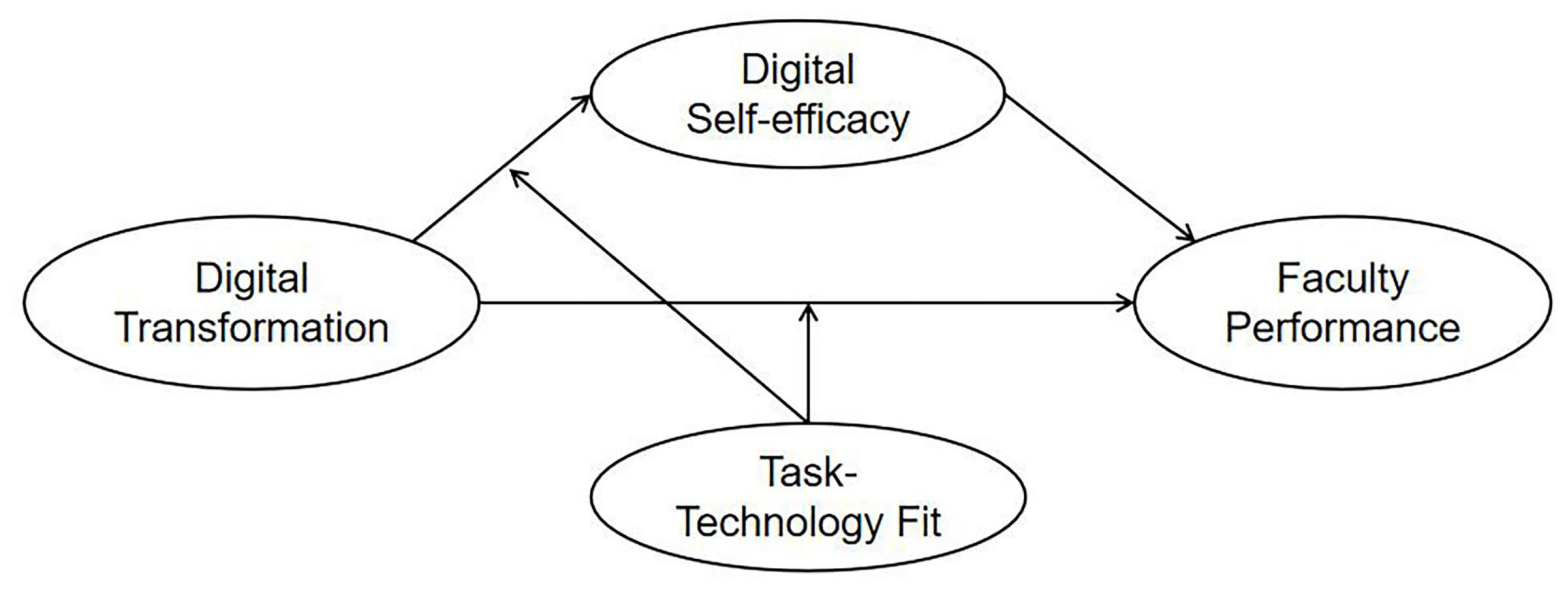
Research hypothesis model.

This study employs Self-Determination Theory to reveal the intrinsic psychological motivation process through which faculty translate digital transformation into enhanced performance. Task-Technology Fit Theory defines the external contextual boundary conditions that determine whether this psychological process can be successfully activated. By integrating both theories, this study addresses two fundamental questions: How does digital transformation affect faculty performance, and under what conditions is this influence most effective? Based on the theoretical background and research hypotheses outlined above, a conceptual model is proposed to delineate the impact of digital transformation in higher education on faculty performance, as illustrated in Figure 1.

## 3 Methods

### 3.1 Participants and procedure

This study focuses on faculty members in Shandong Province, China. In terms of the scale of higher education, Shandong Province ranks among the top four provinces in China regarding the number of existing higher education institutions, students, and faculty numbers. Moreover, Shandong’s higher education system boasts diverse types, including research universities as well as specialized institutions such as science and engineering, teacher training, agriculture, and medical colleges. Additionally, Shandong Province is deeply implementing the strategy of digital education reform, which can better reflect the changes brought about by digital transformation.

This study employed convenience sampling and conducted an online survey among faculty in Shandong Province in August 2025, using the Wenjuanxing online questionnaire platform. Participants could voluntarily choose to enter the survey link after reading the informed consent form. A total of 525 questionnaires were collected. After excluding invalid responses such as incomplete completion, irregular answers, insufficient response time, and logical inconsistencies, 482 valid questionnaires remained, achieving a 91.8% validity rate. Among the participants, 217 (45%) were from undergraduate institutions and 265 (55%) from vocational colleges. In terms of disciplinary background, 279 (57.9%) were from natural sciences and 203 (42.1%) from humanities and social sciences. The sample included 235 (48.8%) male and 247 (51.2%) female faculty members. With regard to teaching experience, 134 (27.8%) had 0–4 years, 135 (28%) had 5–9 years, 133 (27.6%) had 10–19 years, and 80 (16.6%) had over 20 years. As for title, 195 (40.5%) held junior level, 162 (33.6%) were at the middle level, 102 (21.2%) at the associate senior level, and 23 (4.8%) at the full senior level. The details are shown in in Table 1.

**Table 1:** General demographic characteristics of the survey respondents (*n* = 482).

Descriptor	Category	*n*	%
Gender	Male	235	48.8%
	Female	247	51.2%
Age	≤29 years	143	29.7%
	30–39 years	135	28.0%
	40–49 years	128	26.6%
	≥50 years	76	15.8%
Type of Institution	Undergraduate universities	217	45.0%
	Vocational College	265	55.0%
Discipline	Natural Sciences	279	57.9%
	Humanities and Social Sciences	203	42.1%
Teaching Experience	≤4 years	134	27.8%
	5–9 years	135	28.0%
	10–19 years	133	27.6%
	≥20 years	80	16.6%
Title	Junior	195	40.5%
	Middle	162	33.6%
	Deputy high	102	21.2%
	High	23	4.8%

### 3.2 Measures

#### 3.2.1 Faculty performance (FP)

The study employs the 8-item faculty performance scale developed by Chen (2016), comprising two dimensions: research and social service performance (5 items, e.g., I invest significant effort in research and have achieved high-level awards for research outcomes) and teaching performance (3 items, e.g., I proactively enrich course content by using advanced teaching materials and connecting theory with practice to ensure instructional quality). All items were rated on a 6-point Likert scale (1 = “strongly disagree,” 6 = “strongly agree”), and the higher the score, the better the faculty performance. The Cronbach’s α coefficient of 0.920 validates the scale’s reliability, while confirmatory factor analysis (CFA) reveals structural validity through model fit indices: χ^2^/df = 1.097, RMSEA = 0.017, RMR = 0.033, CFI = 0.992, GFI = 0.991, NFI = 0.995, demonstrating the scale’s good structural validity.

#### 3.2.2 Digital transformation (DT)

The digital transformation was measured with the 24-item scale developed by Begicevic Redjep et al. (2021). The scale consists of five dimensions, namely: Information and Communications Technology (ICT) Infrastructure (4 items, e.g., The institution provides ICT equipment for educational staff), ICT Culture (5 items, e.g., Educational staff can access ICT resources), ICT in Learning and Teaching (6 items, e.g., Whether ICT is used in teaching and learning), Planning, Management and Leadership (4 items, e.g., Does the institution have a vision, strategic guidelines and objectives of ICT integration), Development of digital competences (5 items, e.g., Does the institution have a plan for digital competences development). All items were rated on a 4-point Likert scale (1 = “Almost none,” 4 = “Almost all”), and the higher the score, the higher the maturity of digital transformation. The Cronbach’s α coefficient of 0.964 validates the scale reliability, while CFA reveals structural validity through model fit indices: χ^2^/df = 1.642, RMSEA = 0.037, RMR = 0.057, CFI = 0.989, GFI = 0.937, NFI = 0.972, demonstrating the scale’s good structural validity.

#### 3.2.3 Digital self-efficacy (DSE)

The digital self-efficacy was measured with the 25-item scale developed by Ulfert-Blank and Schmidt (2022). The scale consists of five dimensions, namely: Information and Data Literacy (3 items, e.g., I could distinguish between correct and incorrect digital information), Communication and Collaboration (8 items, e.g., I could share information and data with others digitally), Digital Content Creation (4 items, e.g., I could create digital content), Safety (5 items, e.g., I could protect my digital devices from unwanted access), Problem-solving (5 items, e.g., I could identify technical problems when using digital environments). All items were rated on a 6-point Likert scale (1 = “strongly disagree,” 6 = “strongly agree”), and the higher the score, the higher of the digital self-efficacy. The Cronbach’s α coefficient of 0.957 validates the scale reliability, while CFA reveals structural validity through model fit indices: χ^2^/df = 1.764, RMSEA = 0.040, RMR = 0.053, CFI = 0.985, GFI = 0.930, NFI = 0.965, demonstrating the scale’s good structural validity.

#### 3.2.4 Task-technology fit (TTF)

The task-technology fit was measured with the 6-item scale developed by Howard and Rose (2019), which contains one dimension (e.g., Digital technology is suitable for your task). All items were rated on a 7-point Likert scale (1 = “strongly disagree,” 7 = “strongly agree”), and the higher the score, the higher degree of the task-technology fit. The Cronbach’s α coefficient of 0.965 validates the scale reliability, while CFA reveals structural validity through model fit indices: χ^2^/df = 1.947, RMSEA = 0.044, RMR = 0.035, CFI = 0.997, GFI = 0.988, NFI = 0.995, demonstrating the scale’s good structural validity.

### 3.3 Control variables

Existing literature indicates that gender, experience, age, and type of educational institution serve as significant predictors of teacher performance (Hanif et al., 2011; Layek and Koodamara, 2024). Furthermore, teaching effectiveness and outcomes differ according to academic title (Cadez et al., 2017), while variations in teaching achievements (Neumann, 2001) and academic achievements (Brint et al., 2012) are also observed across disciplines. Differences across demographic variables were examined separately. T-test results indicated no significant differences in faculty performance by gender (*p* = 0.309) or discipline (*p* = 0.794). One-way ANOVA showed that neither age (*p* = 0.134) nor title (*p* = 0.188) had a significant effect on faculty performance. However, teaching experience (*p* < 0.001) and institution type (*p* < 0.001) were found to have a significant influence on performance. Accordingly, teaching experience and institution type were included as control variables in the analysis. These variables were coded as follows: Teaching experience (1 = ≤ 4 years, 2 = 5–9 years, 3 = 10–19 years, 4 = ≥ 20 years); Institution type (1 = undergraduate universities, 2 = vocational college).

### 3.4 Analytical strategy

In this study, SPSS 27.0 was used for descriptive analysis, analysis of variance, correlation analysis, and common method bias test, AMOS 26.0 was used for confirmatory factor analysis, and PROCESS was used for hypothesis testing.

## 4 Results

### 4.1 Common method biases

To reduce the influence of common method bias resulting from participants’ self-assessment, several procedural controls were implemented: the order of questions was randomized, positive and negative items were balanced, and different Likert scales were employed. During the survey, participants were assured that their responses would remain anonymous and that their data would be kept confidential. The collected data were subjected to Harman’s single-factor analysis to test for common method bias. The results yielding a KMO = 0.957 (*p* < 0.001), indicating that suitability for factor analysis. Exploratory factor analysis was conducted on all the measurement items. The results showed that 13 factors were extracted without rotation, and the first common factor accounted for 39.455% of the total load, which was below the discriminant criterion of 40%, indicating that no significant common method bias was present (Podsakoff et al., 2003).

### 4.2 Descriptive statistics and correlations

Descriptive statistics and correlation analyses were performed on the data. Spearman’s correlation analyses were conducted on demographic variables, and Pearson’s correlation tests were performed on the four main variables. The results are presented in Table 2. Since demographic variables were categorical in the questionnaire, means and standard deviations are not presented in the table. Significant correlations (*p* < 0.01) were identified between all four main variables: FP was significantly and positively correlated with DT (r = 0.627), TTF (r = 0.228), and DSE (r = 0.664). DT was significantly and positively correlated with TTF (r = 0.326) and DSE (r = 0.196). TTF and DSE (r = 0.196) were significantly positively correlated. The correlations between the variables provide a preliminary basis for subsequent tests of mediating effects.

**Table 2:** Descriptive statistics and correlations.

Variable	M	SD	Gender	Age	InstType	Discipline	TeachExp	Title	FP	DT	TTF	DSE
Gender	-	-	--									
Age	-	-	0.003	--								
InstType	-	-	0.018	−0.251*	--							
Discipline	-	-	0.555**	0.028	0.029	--						
TeachExp	-	-	0.014	0.793**	−0.133*	0.019	--					
Title	-	-	0.008	0.803**	−0.265**	0.027	0.890**	--				
FP	3.737	1.326	0.046	0.123*	−0.355**	0.012	0.574**	0.162*	0.904			
DT	3.689	1.280	0.05	0.046	−0.105	0.042	0.112*	0.192*	0.627**	0.909		
TTF	4.286	1.951	−0.042	0.097	−0.146*	0.126*	0.214**	0.256**	0.228**	0.326**	0.905	
DSE	3.795	1.165	0.034	0.631**	−0.301**	0.057	0.649**	0.660**	0.664**	0.696**	0.179**	−0.896

**p < 0.01, *p < 0.05. Diagonal values are square roots of AVE values.

### 4.3 Convergent and discriminant validity tests

The standardized factor loadings (β) of all measurement items were greater than 0.7 (FP: 0.891–0.931, DT: 0.877–0.951, TTF: 0.898–0.922, and DSE: 0.876–0.942), and all were statistically significant (*p* < 0.001), indicating that the measurement items were strongly correlated with and representative of their respective constructs. By calculating the average extracted variance (AVE) and combined reliability (CR), it was found that the AVE value of each construct was greater than 0.5 and the CR was greater than 0.7, indicating that the constructs had high internal consistency and convergent validity (Fornell and Larcker, 1981). The specific results are shown in Table 3.

**Table 3:** Results of convergent validity.

Variables	Measurement item	B	β	S.E.	C.R.	*p*	CR	AVE
FP	FP1	1.000	0.931	/	/	/	0.973	0.818
	FP2	0.959	0.893	0.029	33.332	<0.001		
	…	…	…	…	…	<0.001		
	FP8	0.988	0.902	0.034	28.708	<0.001		
DT	DT1	1.000	0.942	/	/	/	0.991	0.827
	DT2	0.964	0.909	0.027	35.877	<0.001		
	…	…	…	…	…	<0.001		
	DT24	0.985	0.905	0.028	35.457	<0.001		
TTF	TTF1	1.000	0.922	/	/	/	0.965	0.819
	TTF2	0.961	0.899	0.029	33.139	<0.001		
	…	…	…	…	…	<0.001		
	TTF6	0.998	0.903	0.030	33.610	<0.001		
DSE	DSE1	1.000	0.921	/	/	/	0.990	0.804
	DSE2	0.979	0.882	0.034	29.097	<0.001		
	…	…	…	…	…	<0.001		
	DSE25	0.952	0.894	0.028	34.364	<0.001		

The test for discriminant validity between constructs was performed and the results are shown in Table 2. The values on the diagonal are the squared differences of the AVE values of the constructs and the remaining values are the correlation coefficients between the constructs. The results show that the correlation coefficients between the constructs are significantly smaller than the square root of the AVE of the row or column in which they are located. This indicates good discriminant validity (Fornell and Larcker, 1981).

### 4.4 Hypothesis testing and path analysis

#### 4.4.1 Mediating effect

Path analysis was conducted with faculty performance as the dependent variable, digital transformation as the independent variable, digital self-efficacy as the mediator, and teaching experience and institution type as control variables. PROCESS model 4 was employed, and the bias-corrected percentile Bootstrap method was used for 5,000 resamples at a 95% confidence interval. The results are shown in Table 4. The standardized total effect value of digital transformation on faculty performance was 0.420 (*p* < 0.001), and the 95% CI [0.317, 0.522] did not include 0, indicating that digital transformation in higher education has a significant positive contribution to faculty performance, and Hypothesis 1 was verified.

**Table 4:** Path analysis results.

Paths		Effects	*p*	95% CI		Relative mediation effect (%)
				Lower	Upper	
DT → DSE		0.429	<0.001	0.347	0.511	/
DSE → FP		0.455	<0.001	0.350	0.560	/
DT → FP	Direct path: DT → FP	0.225	<0.001	0.118	0.330	53.6%
	Indirect path: DT → DSE → FP	0.195	<0.001	0.132	0.264	46.4%
	Total effect	0.420	<0.001	0.317	0.522	100

The standardized effect of digital transformation on digital self-efficacy was 0.429 (*p* < 0.001), and the 95% CI [0.347, 0.511] did not include 0, indicating that digital transformation has a significant positive facilitating effect on digital self-efficacy. The standardized effect of digital self-efficacy on faculty performance was 0.455 (*p* < 0.001), and the 95% CI [0.350, 0.560] did not include 0, indicating that digital self-efficacy has a significant positive contribution to faculty performance. When digital self-efficacy was treated as the mediating variable, the standardized indirect path effect of digital transformation → digital self-efficacy → faculty performance was 0.195 (*p* < 0.001), and the 95% CI [0.132, 0.264] did not include 0. These results indicate that digital self-efficacy plays a significant mediating role in the relationship between digital transformation and faculty performance.

The standardized direct path effect of digital transformation on faculty performance was 0.225 (*p* < 0.001), and the 95% CI [0.118, 0.330] did not include 0. This indicates that even after introducing mediating variables, digital transformation in higher education still exerts a significant positive effect on faculty performance. This suggests that digital self-efficacy partially mediates this relationship. The effect values from direct and indirect paths accounted for 53.6 and 46.4%, respectively.

#### 4.4.2 Moderating effects

Path analysis was conducted using PROCESS Model 8, with faculty performance as the dependent variable, digital transformation as the independent variable, digital self-efficacy as the mediator, task-technology fit as the moderator, and teaching experience and institution type as control variables. The bias-corrected percentile Bootstrap method was applied with 5,000 resamples at a 95% confidence interval, and the results are present in Table 5.

**Table 5:** Results of moderated effects.

Model	Dependent variable	Independent Variables	Coeff.	S.E	t	*p*	LLCI	ULCI
Model 1	DSE	TeachExp	0.234	0.045	5.222	<0.001	0.146	0.322
		InstType	0.038	0.071	0.527	0.598	−0.103	0.178
		DT	0.444	0.038	11.577	<0.001	0.368	0.519
		TTF	−0.006	0.018	−0.356	0.722	−0.041	0.028
		DT × TTF	0.157	0.014	11.268	<0.001	0.130	0.185
Model 2	FP	TeachExp	0.122	0.056	2.172	0.030	0.012	0.233
		InstType	−0.220	0.087	−2.531	0.012	−0.392	−0.049
		DT	0.302	0.053	5.705	<0.001	0.198	0.406
		DSE	0.240	0.056	4.287	<0.001	0.130	0.350
		TTF	0.050	0.022	2.315	0.021	0.008	0.092
		DT × TTF	0.164	0.019	8.570	<0.001	0.126	0.202

According to the test results, after including task-technology fit as a moderator, digital transformation remained significant positive predictor of digital self-efficacy (B = 0.444, *p* < 0.001), whereas task-technology fit has no significant effect on digital self-efficacy (B = -0.006, *p* = 0.722). However, the interaction term of digital transformation and task-technology fit had a significant positive effect on digital self-efficacy (B = 0.157, *p* < 0.001), indicating that task-technology fit has a significant positive moderating effect between digital transformation and digital self-efficacy. As a result, hypothesis H3b was supported.

In addition, after including the moderating variable, digital transformation (B = 0.302, *p* < 0.001) and digital self-efficacy (B = 0.240, *p* < 0.001) remained significant positive predictor of faculty performance. There was also a significant positive effect of task-technology fit on faculty performance in this model (B = 0.050, *p* = 0.021). The interaction between digital transformation and task-technology fit had a significant effect on faculty performance (B = 0.164, *p* < 0.001), indicating that task-technology fit positively moderates the relationship between digital transformation and faculty performance. Hypothesis H3a was supported.

To further explore the role of digital transformation under different levels of task-technology fit, one standard deviation was added to and subtracted from the mean of task-technology fit to form high (M + 1SD) and low (M-1SD) TTF group, respectively. A simple slope analysis was then conducted.

**Figure 2:**
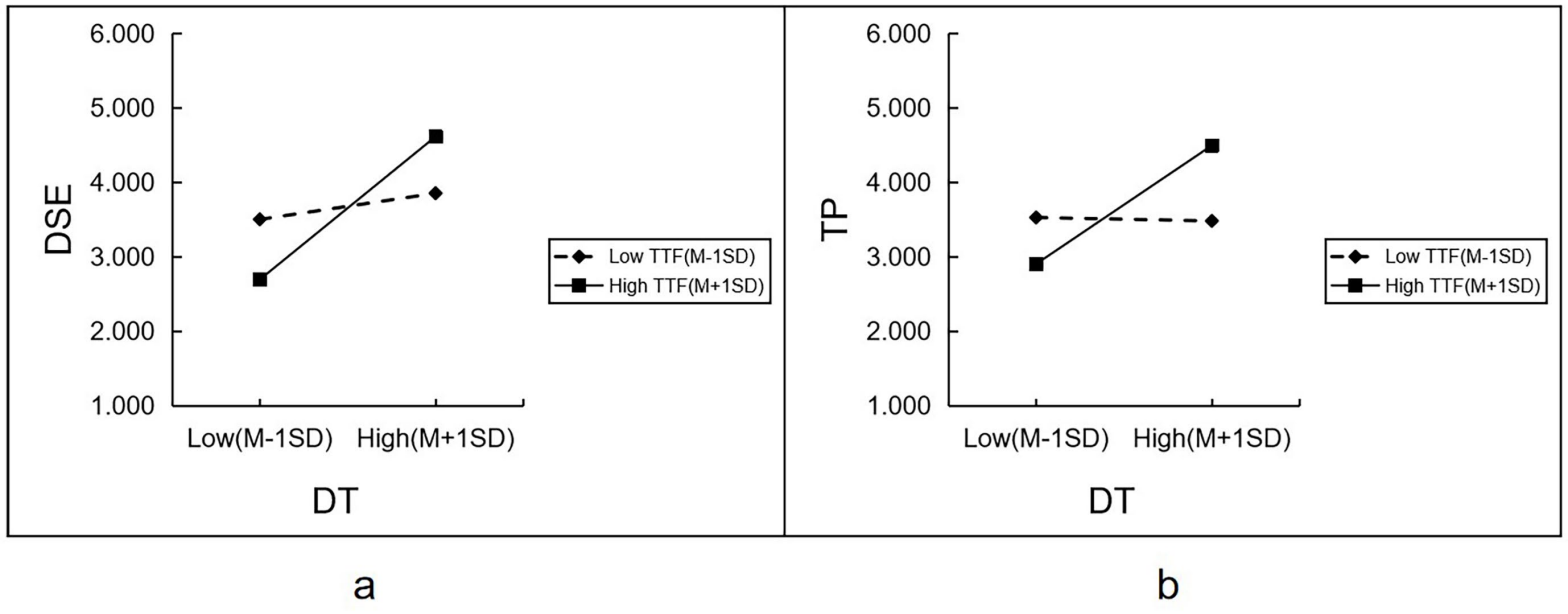
Simple slope analysis.

As can be seen in Figure 2a, the effect of digital transformation on digital self-efficacy varies according to the level of task-technology fit. The positive effect of digital transformation on digital self-efficacy is higher under task technology fit (B = 0.751, t = 15.977, *p* < 0.001) than under low task technology fit (B = 0.137, t = 2.916, *p* = 0.004).

According to Figure 2b, the impact of digital transformation on faculty performance varies according to the level of task-technology fit. Under low task-technology fit, the effect of digital transformation on faculty performance is nonsignificant (B = 0.018, t = 0.309, *p* = 0.757), whereas, under high task-technology fit, digital transformation significantly positively affected faculty performance (B = 0.622, t = 8.756, *p* < 0.001).

#### 4.4.3 Moderated mediated effects

In testing the moderated mediation model, the results showed that the moderated mediation effect index was 0.038, with a 95% confidence interval of [0.012, 0.065], which does not include 0, indicating that the indirect effect of DT → DSE → FP is positively moderated by task-technology fit. The moderated effect values are shown in Table 6. The 95% CI for the mediating path DT → DSE → FP, which does not include 0, is significant at all three different levels of task-technology fit. However, the indirect effect of digital transformation affecting faculty performance through digital self-efficacy is stronger at higher levels of task-technology fit.

**Table 6:** Results of moderated mediation effect.

TTF	Effect	BootSE	BootLLCI	BootULCI
M-1SD	0.033	0.018	0.003	0.071
M	0.106	0.036	0.036	0.179
M + 1SD	0.180	0.060	0.059	0.300

## 5 Discussion

First, digital transformation in higher education significantly enhances faculty performance. In the era of educational digitization, faculty serve as key users of digital technologies. Previous studies have identified various digital tools impacting teaching and research outcomes, such as intelligent tutoring systems (Lin et al., 2023), virtual reality (VR) technology (Marks and Thomas, 2022), and remote learning solutions (Abaci et al., 2021). However, in practice, digital technology does not play a separate role. Other factors such as digital culture, digital leadership, and digital infrastructure can all affect the impact of digital technology on performance. This study therefore integrates these external digital-related elements into a unified framework—digital transformation—as an environmental variable to examine its impact on faculty performance. The results indicate that digital transformation significantly promotes faculty performance, the greater the maturity of digital transformation, the more effectively it translates digital technologies into faculty performance. This finding aligns with previous research showing that familiarity with technology and robust digital infrastructure contribute to improved digital teaching quality (Fütterer et al., 2023; Wohlfart et al., 2021) and reduce technology-related anxiety (Fernández-Batanero et al., 2021). In the technological era, managers must incorporate achievement orientation and performance enhancement into their leadership practices. These results provide a theoretical basis for higher education administrators to improve faculty performance by enhancing digital transformation in the context of educational digitization.

Second, digital self-efficacy serves as a mediator in the relationship between digital transformation and faculty performance. This conclusion reveals the potential mechanism by which digital transformation affects faculty performance, namely that mature digital transformation can enhance faculty’s digital self-efficacy, thereby promoting faculty performance. This conclusion confirms the hypothesis in self-determination theory that people’s level of self-determination is influenced by external environmental support (Ryan and Deci, 2000), as well as the close relationship between self-efficacy and performance (Szulawski et al., 2021). Previous studies have shown that mature device software (Yu et al., 2017) and a digital cultural atmosphere (Velyako and Musa, 2024) can help cultivate teachers’ digital self-efficacy, and teachers with higher digital self-efficacy are better able to apply digital technology to their work (Cosby et al., 2023). This study treats digital transformation as a “demand-supportive environment,” applying self-determination theory to the context of institutional change in higher education, thereby enriching the application of self-determination theory within technology-driven organizations. The results fill the gap in research on the relationship between digital transformation and digital self-efficacy, confirm the predictive effect of digital self-efficacy on faculty performance, and provide a more comprehensive understanding of the interplay among digital transformation, digital self-efficacy, and faculty performance. This finding provides a new perspective for understanding how digital transformation affects faculty performance.

Third, task-technology fit strengthens the mediating effect of digital self-efficacy in the relationship between digital transformation and faculty performance. Previous studies have predominantly treated task-technology fit as an independent variable to investigate its effects on technology adoption intention (Wang et al., 2024) or satisfaction (Alturki and Aldraiweesh, 2023). In this study, TTF is introduced as a moderator in the pathway from digital transformation to faculty performance. This approach breaks from the traditional paradigm where TTF directly influences outcome variables, thereby expanding the application scope of TTF theory. The result indicates that under conditions of high task-technology fit, digital transformation can positively promote faculty performance. This result is consistent with the previous research that high task-technology fit can have a positive impact on users and improve productivity (Yuce et al., 2019). Moreover, the mediating effect of digital self-efficacy is stronger under high TTF and weaker under low TTF. This further confirms that higher TTF facilitates teachers’ acquisition of digital self-efficacy, thereby enhancing performance (Dahri et al., 2024). This finding suggests that for digital technology to more effectively enhance teacher performance, it requires not only environmental support (digital transformation) and psychological motivation (digital self-efficacy), but also task-technology alignment. It also informs administrators that advanced technology is not necessarily effective technology. When introducing new technologies during digital transformation, task needs analysis must be conducted first to ensure the technology aligns closely with faculty’s core tasks. The results of this study not only enrich the research perspective of TTF theory, but also provide a theoretical basis for how higher education organizations can use digital transformation to improve faculty performance.

Fourth, this study integrates Self-determination theory and task-technology fit theory, and provides theoretical basis for understanding the transition from digital transformation to faculty performance from two dimensions: human internal motivation and technology external context. Self-Determination Theory highlights the importance of environments that satisfy individuals’ competence needs. In this study, digital transformation is conceptualized as a “need-supportive environment,” specifying task-technology fit as a key contextual prerequisite for satisfying competence needs within digital settings. By translating self-determination theory to the context of digital change in higher education, this study enriches its application within technology-driven organizations. Furthermore, traditional research on task-technology fit has predominantly focused on its direct consequences for usage behavior or performance. Our study shifts the role of task-technology fit to the stage of psychological motivation formation. Results demonstrate that TTF not only influences “how to do” but also impacts “how to think,” revealing a new role for TTF as a “cognitive catalyst” and enriching its theoretical research perspective.

## 6 Limitations and future directions

This study has several limitations that should be acknowledged. First, the sample was limited to faculty in Shandong Province, which may restrict the generalizability of the findings to other regions due to geographical and cultural variations. Given the uneven development of higher education in China (Wu et al., 2020), disparities exist among different regions in terms of educational resources, teacher beliefs, and work pressure (Sang et al., 2009). Future research could enhance the generalizability through cross-cultural or cross-regional studies. Second, data collection relied on self-reports from respondents, which may introduce common method bias. Although procedural controls were applied and Harman’s single-factor test was passed, common method bias cannot be completely eliminated, and social desirability bias may still be present. Previous research has confirmed the influence of social desirability tendencies on self-reported data from educators (Kopcha and Sullivan, 2007). To improve robustness and validity, future studies should employ multiple data collection methods, such as student assessments (Scherer and Gustafsson, 2015) and observational methods (Martinez et al., 2016). Additionally, the cross-sectional design limits the ability to make causal interpretations. Longitudinal studies examining changes over extended periods or under varying conditions would provide clearer evidence of causality.

## 7 Conclusion

This study established a moderated mediation model using digital transformation as the independent variable, digital self-efficacy as the mediating variable, task-technology fit as the moderating variable, and faculty performance as the dependent variable. The findings revealed that digital transformation significantly positively predicts faculty performance. Digital self-efficacy partially mediates the relationship between digital transformation and faculty performance. Task-technology fit positively enhances the impact of digital transformation on faculty performance, and when the level of task-technology fit is high, the indirect effect of digital transformation on faculty performance through digital self-efficacy is stronger.

## Data Availability

The raw data supporting the conclusions of this article will be made available by the authors, without undue reservation.
